# Model Selection in Time Series Studies of Influenza-Associated Mortality

**DOI:** 10.1371/journal.pone.0039423

**Published:** 2012-06-20

**Authors:** Xi-Ling Wang, Lin Yang, King-Pan Chan, Susan S. Chiu, Kwok-Hung Chan, J. S. Malik Peiris, Chit-Ming Wong

**Affiliations:** 1 School of Public Health, The University of Hong Kong, Hong Kong Special Administrative Region, China; 2 Department of Pediatrics and Adolescent Medicine, The University of Hong Kong, Hong Kong Special Administrative Region, China; 3 Department of microbiology, Queen Mary Hospital, Hong Kong Special Administrative Region, China; 4 The University of Hong Kong Pasteur Research Center, Hong Kong Special Administrative Region, China; National University of Singapore, Singapore

## Abstract

**Background:**

Poisson regression modeling has been widely used to estimate influenza-associated disease burden, as it has the advantage of adjusting for multiple seasonal confounders. However, few studies have discussed how to judge the adequacy of confounding adjustment. This study aims to compare the performance of commonly adopted model selection criteria in terms of providing a reliable and valid estimate for the health impact of influenza.

**Methods:**

We assessed four model selection criteria: quasi Akaike information criterion (QAIC), quasi Bayesian information criterion (QBIC), partial autocorrelation functions of residuals (PACF), and generalized cross-validation (GCV), by separately applying them to select the Poisson model best fitted to the mortality datasets that were simulated under the different assumptions of seasonal confounding. The performance of these criteria was evaluated by the bias and root-mean-square error (RMSE) of estimates from the pre-determined coefficients of influenza proxy variable. These four criteria were subsequently applied to an empirical hospitalization dataset to confirm the findings of simulation study.

**Results:**

GCV consistently provided smaller biases and RMSEs for the influenza coefficient estimates than QAIC, QBIC and PACF, under the different simulation scenarios. Sensitivity analysis of different pre-determined influenza coefficients, study periods and lag weeks showed that GCV consistently outperformed the other criteria. Similar results were found in applying these selection criteria to estimate influenza-associated hospitalization.

**Conclusions:**

GCV criterion is recommended for selection of Poisson models to estimate influenza-associated mortality and morbidity burden with proper adjustment for confounding. These findings shall help standardize the Poisson modeling approach for influenza disease burden studies.

## Introduction

Numerous studies have demonstrated that influenza causes substantial burden on mortality and morbidity [1]–[3]. Reliable estimates for disease burden associated with influenza in the community are essential for public health policy-making. However the case numbers of influenza infections derived from medical records grossly underestimated the true burden [4]. During 2001 to 2009 there were only 138 deaths registered in Hong Kong with underlying cause of influenza infection [5]. Underreporting of influenza cases was due to the fact that influenza infections usually caused relatively mild symptoms and many infected people did not seek medical care from hospital or clinic. Among outpatients and inpatients with influenza-like illness, few were tested for influenza to get confirmed diagnoses. Even for those with laboratory confirmed infections, influenza was rarely recorded as underlying cause of death on their death certificates. Several statistical models have been used to quantify the disease burden attributable to influenza [6]. Among these models, Poisson regression models have become increasingly popular in recent years [7]–[9]. Unlike most of the other models, the Poisson model does not require clear seasonality of influenza to define influenza epidemic and non-epidemic periods. Therefore, it is particularly suitable for tropical and subtropical regions where influenza seasonality is less clear and influenza viruses could be circulating throughout the whole year.

Another advantage of the Poisson model lies in its ability to adjust for multiple seasonal confounders simultaneously. There are two types of confounders that are often considered in Poisson models: measured confounders, such as meteorological factors, circulation of other respiratory pathogens and air pollution [10]; and unmeasured confounders, such as seasonal change in host susceptibility and health seeking behavior [11]. However, over-adjustment of confounders may result in underestimation of true effects, as some variations caused by influenza were allocated to confounders. Likewise, inadequate adjustment could lead to residual confounding that causes spurious association between influenza proxy variable (such as proportions of specimens testing positive for influenza) and health outcome of mortality or hospitalization. Therefore, proper adjustment of confounders is critical for obtaining reliable estimates of influenza-associated disease burden. Previous studies using Poisson models adjusted for unmeasured confounders by incorporating sinusoidal pairs [12]–[15] or a smoothing function of time trend into the Poisson model [8], [16], [17]. However, few studies has properly discussed on how to determine the adequacy of adjustment for seasonal confounders in the model. Here we conducted a simulation study with the aim to compare the performance of several commonly adopted model selection criteria, in terms of selecting the best-fit Poisson model with adequate adjustment for confounders. Four model selection criteria were considered in this study: quasi Akaike information criterion (QAIC), quasi Bayesian information criterion (QBIC), partial autocorrelation functions of residuals (PACF), and generalized cross-validation (GCV).

## Methods

### Data

We obtained weekly all-cause mortality data from 1998 to 2008 from the Census & Statistics Department, and daily meteorological data of temperature and relative humidity from the Hong Kong Observatory. Daily concentrations of air pollutants nitrogen dioxide (NO_2_), sulfur dioxide (SO_2_), ozone (O_3_) and particulate matters with diameter less than 10 µm (PM_10_) were obtained from the Environmental Protection Department. Weekly numbers of specimens positive for influenza and respiratory syncytial virus (RSV) as well as total numbers of tested specimens were obtained from the microbiology laboratory of Queen Mary Hospital. Influenza virology data of this single laboratory have been demonstrated representative of the virus activity in the entirety of Hong Kong [16].

### Mortality Data Simulation

We performed a simulation study by generating mortality data from a Poisson model with adjustment for over-dispersion [18]. This model is similar to the models used in our previous studies on influenza-associated mortality and morbidity [17], in which an influenza proxy variable is added to assess influenza effects. To derive a proper estimate for influenza-associated mortality or morbidity, it is important to adjust for confounding to separate the effect of influenza from those of other seasonal factors. Co-circulation of RSV, together with two meteorological factors of temperature and humidity, are adjusted for as confounders in this study given their association with both health outcomes and influenza [19], [20]. Weekly concentrations of four major ambient air pollutants are also included as confounders based on recent findings on the association between influenza virus and ambient air pollutants [21]. Unmeasured confounding is adjusted for by including the long-term and seasonal trends of outcome variables. A typical model was as follows:
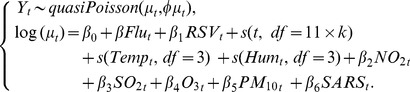
(1)
*Y_t_* denotes the weekly number of all-cause deaths which was assumed to follow a Poisson distribution with an over-dispersion parameter *φ* [22]. *Flu_t_* and *RSV_t_* denote the proxy variables for influenza and RSV, which are weekly proportions of specimens testing positive for influenza or RSV. Three natural cubic spline smoothing functions of s(t, *df = 11*×*k*), s(*Temp_t_*) and s(*Hum_t_*) are added to adjust for time (t = 1,2,…,574), weekly mean temperature (*Temp_t_* ) and relative humidity (*Hum_t_*), where the degrees of freedom (*df*) of time *k* ranges from 1 to 10 per year. We used a natural cubic spline with fixed degrees of freedom so that the locations of knots were evenly distributed [23]. *NO_2_*_t_, *SO_2_*_t_, *O_3_*_t_ and *PM_10_*_t_ denote the weekly mean concentrations of four air pollutants, respectively. To adjust for the increased mortality during the outbreak of Severe Acute Respiratory Syndrome (SARS) in 2003, we added into the model a dummy variable *SARS* for the SARS period of week 1–30 of year 2003.

Before simulation, we first estimated the coefficients of model (1) by fitting it to the all-cause mortality data during 1998 to 2008 in Hong Kong. The degrees of freedom were fixed to three for weekly temperature and humidity based on our previous experience [17] and to one per year for long term and seasonal trends. Mean mortality for week 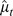
was then predicted from this fitted model with the β coefficient for influenza variable *Flu_t_* fixed to 0.33 (i.e. mortality increasing 3.3% when the influenza positive proportion increases 10%). The over-dispersion parameter 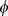
was also derived from this model. Because there was no statistical package available for data simulation based on the over-dispersed Poisson distribution, we simulated 500 mortality datasets by assuming that mortality followed a negative binomial distribution, i.e. 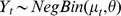 when 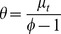 [24]. Given the uncertainty in degrees of freedom for unmeasured seasonal confounders, we repeated the above simulation process with the degrees of freedom of s(*t*, *df*) changing from 1 to 2,3,…, 10 per year. Hence, we got a total of 5000 weekly mortality datasets and 500 for each fixed degrees of freedom for *t*.

### Model Selection Criteria Comparison

We then applied Model (1) with degrees of freedom varying from 1–10 per year for s(*t*,*df*) to each set of 500 simulated data), and selected the best-fit model with the minimal value for each of the following model selection criteria:

1. Quasi-Akaike information criterion (QAIC) [25]:
    QAIC(*df*) =  (−2(maximum log-likelihood)/over-dispersion   parameter) +2*df*
2. Quasi-Bayesian information criterion (QBIC) [26]:
    QBIC(*df*) =  (−2(maximum log-likelihood)/over-dispersion   parameter) + log(*n*)×*df*

 where *n* is the number of observations.

1. Residual autocorrelation: the sum of the absolute value of the partial autocorrelation function (PACF) of the residuals up to 5 lag weeks.
2. Generalized cross validation (GCV) [26]:

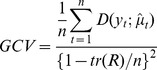
where *tr(R)* is the trace of weighted additive-fit operator corresponding to the last iteration of the local-scoring procedure; *y_t_* is the observed number of death at week *t*; 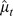
is the predicted number of death at week *t*; 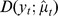 is the deviance of *y_t_* from 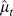; *n* is the number of observations.

We calculated the bias as the average difference between the estimated coefficients of the influenza variable from the best-fit model and the true coefficient of 0.33. Standard error and root-mean-square error (RMSE) were defined as the standard deviation and square root of the mean square error of estimated coefficients, respectively. In this study we took RSME as the primary measure to compare the performance of different model selection criteria, as it could evaluate both accuracy and variation of the estimates [25]. The criterion that obtained the minimal RMSE under the different assumptions of confounding was considered as the best criterion in selecting the model with adequate adjustment for confounders.

To investigate the robustness of our results, we conducted a sensitivity analysis by changing the pre-determined coefficient of influenza variable from 0.33 to 0.1 and 0.5, which were the lower and upper boundaries of influenza effects based on our previous experience. Because a short study period might offer less reliable estimates with large standard error, we did another sensitivity analysis with the data of 2003 to 2008 or those of 2006 to 2008. Given that influenza effects on mortality might lag several weeks behind the increase of influenza activity [15], we separately added the influenza proxy variables up to 3 weeks before (lag 1–3 weeks) into the models to assess any lag effects. All the analyses were conducted using R software (version 2.13.0) [27].

### Application of Models to Empirical Hospitalization Data

We applied the four model selection criteria to an empirical dataset of weekly hospitalization numbers of pediatric patients younger than 18 years. These patients were admitted into two major public hospitals on Hong Kong Island from October 2003 through September 2008, with acute respiratory disease (ARD) listed in the first five discharge diagnoses. These data were retrieved from the computerized database of the Hong Kong Hospital Authority, according to the International Classification of Disease 9^th^ Revision (ICD9) codes of 460−466 or 480−487. Five age groups were considered: 0−1, 1−2, 2−5, 5−10, 10−18 years.
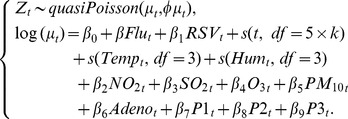
(2)This Poisson model was similar to model (1), except that two proxies for adenovirus (*Adeno_t_*) three types of parainfluenza viruses (*P1_t_, P2_t_, P3_t_*) were added as confounders, because these data were only available after 2003. Influenza-associated hospitalization rates were defined as the difference between the observed and expected hospitalization under the assumption of no circulating influenza viruses. These rates were separately estimated from the best-fit models chosen by each criterion, and the bias and RMSE were calculated by comparing with the observed admission rates of a pediatric cohort of influenza hospitalization cases. As previously described [17], this cohort was composed of all the pediatric patients who were recruited from the same two hospitals and diagnosed with influenza infection by immunofluorescence tests and viral culture. Ethics approval for collecting specimens from pediatric patients was obtained from the Ethics Committee of Li Ka Shing Faculty of Medicine, The University of Hong Kong (EC1880-02).

## Results

**Figure 1:**
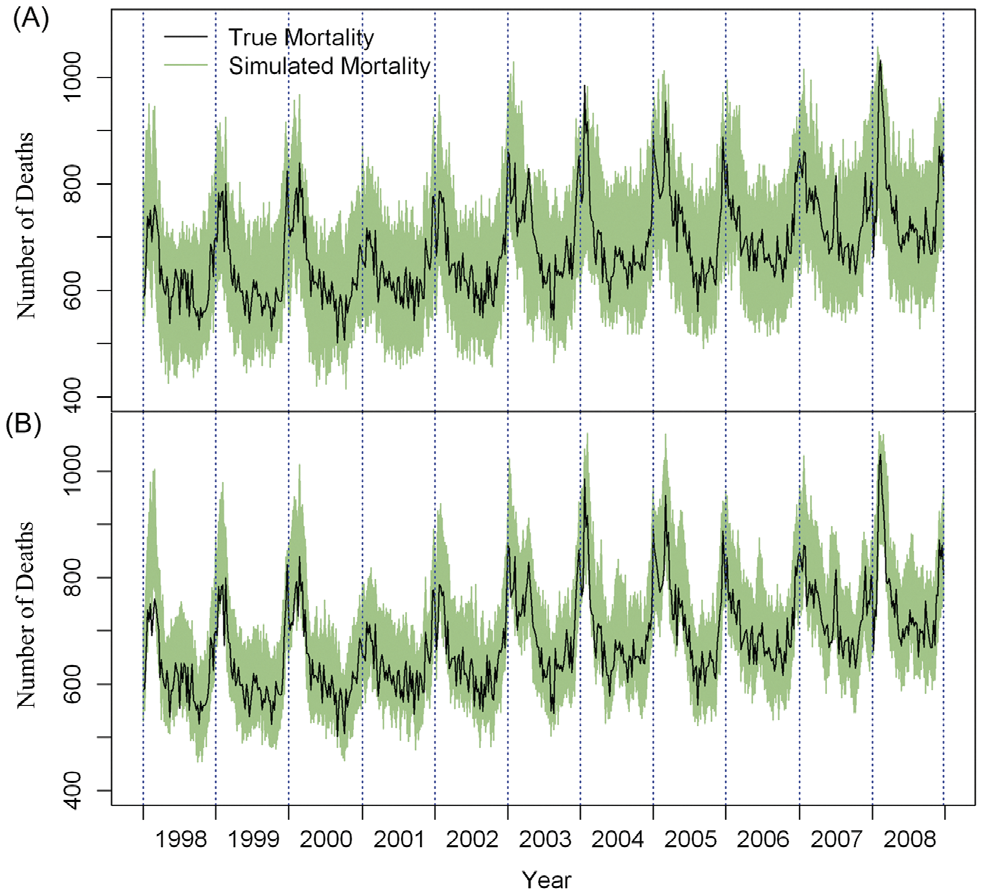
Weekly observed all-cause mortality (black line) and simulated mortality data (green lines). Data were generated (A) under the assumption of low seasonal variation with the degree of freedom for trend set at 1 per year, or (B) under the assumption of high seasonal variation with the degrees of freedom for trend set at 10 per year.

Figure 1 shows the weekly number of deaths simulated under the scenario of low and high seasonal confounding (*df* = 1 and *df* = 10 per year for the seasonal trend smoothing functions). The simulated data fluctuated within the range of 400 to 1100 with a steadily increasing annual trend. As expected, the data simulated under *df* = 10 was rougher and closer to the true mortality than those simulated under *df* = 1 (Figure 1). Overall, the simulated all-cause mortality data were generally comparable with the true mortality data.

**Figure 2:**
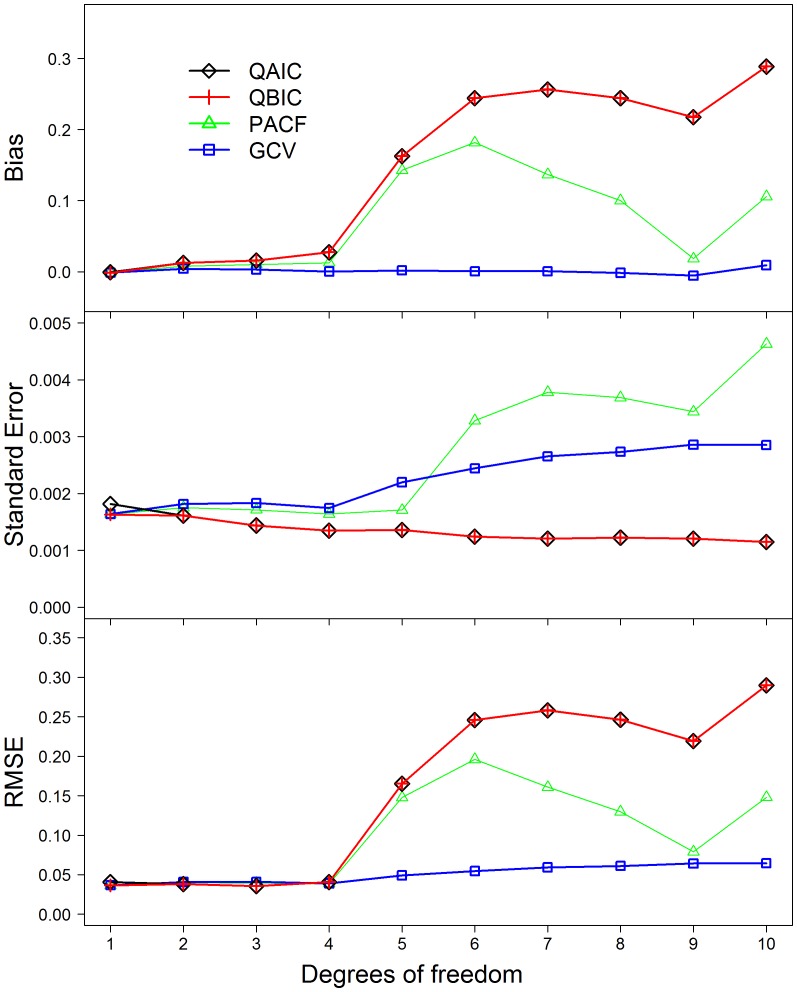
Bias, Standard error and RMSE of influenza coefficients estimated from the best-fit models selected by different criteria. Note: Lines of QAIC and QBIC are overlapping when the degrees of freedom (*df*) range from 2 to 10 per year. Abbreviations: QAIC, quasi-Akaike information criterion; QBIC, quasi-Bayesian information criterion; PACF, partial autocorrelation function; GCV, generalized cross validation; RMSE, root-mean-square error.

Most models overestimated influenza effects with a few exceptions observed for the models selected by the minimal GCV (Figure 2). Estimates tended to have larger biases as the seasonal confounding of the simulated data increased. Overall the models selected by PACF, QAIC and QBIC had the larger biases (ranging from 0.0022 to 0.2909) than did those selected by GCV (ranging from 0.0008 to 0.007) (Figure 2). Standard error of influenza coefficients was comparable between these four criteria, ranging from 0.0012 to 0.0045. RMSE was similar between the models selected by PACF, QAIC or QBIC when the *df* of smoothing functions for time were less than 5 per year, but dramatically increased when the *df* increased to 5 or more per year (Figure 2). The RMSE of GCV criterion remained lower than those of the other three criteria (Figure 2).

Biases and RMSE did not markedly change when the pre-determined coefficient for influenza proxy variable of weekly positive proportions changed from 0.33 to 0.1 and 0.5 (Figure S1). Among the four criteria, GCV still provided the smallest bias and RMSE under the different simulation scenario. Sensitivity analysis of a shorter study period of 2003–2008 or 2006–2008 showed slightly higher RMSE than those from the whole study period, but GCV provided smaller biases and RMSE compared to the other three criteria (Figure S2). In the models with the lag effects of up to 3 weeks, GCV still provided the smallest biases and RMSE for influenza coefficients (Figure S3).

**Figure 3:**
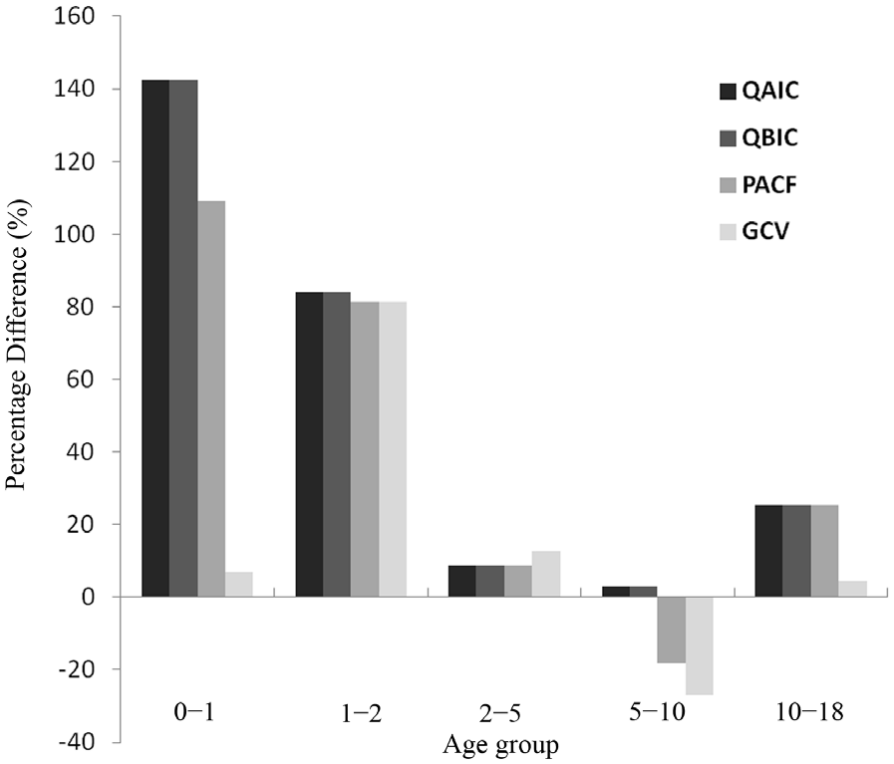
Percentage difference of estimated excess hospitalization rates from the observed admission rates of influenza cases during 2003−2008. Note: Percentage difference = 100%× (estimated excess hospitalization rate – observed rate)/observed rate.

Figure 3 shows the percentage difference between the estimated excess ARD hospitalization rates and directly observed admission rates of influenza cases in the pediatric cohort from 2003 to 2008. For the age groups of 0−1and 10−18 years, the best-fit models selected by GCV provided the estimates closer to the observed rates than did those selected by QAIC, QBIC and PACF (Figure S4). Estimates from the four criteria were comparable for the 1−2 and 2−5 age groups. All the Poisson models respectively selected by the four criteria slightly overestimated the true rates for all the age groups, except that the PACF and GCV criteria provided the estimates smaller than the observed rates in the 5−10 age group. Among the four criteria, GCV had the smallest biases and RMSE, whereas QAIC and QBIC had the largest (Table 1).

**Table 1:** Bias, RMSE of the estimated excess hospitalization rates from the observed hospitalization rates with laboratory confirmed influenza infections.

Criteria	Bias	RMSE
QAIC	46.81	9.55
QBIC	46.81	9.55
PACF	40.66	8.3
GCV	25.93	5.29

Note. QAIC, quasi-Akaike information criterion; QBIC, quasi-Bayesian information criterion; PACF, partial autocorrelation function; GCV, generalized cross validation; RMSE, root-mean-square error.

## Discussion

As underreporting of influenza cases is common in clinical practice, the Poisson modeling approach has been widely accepted in estimating disease burden of influenza [28]. Two recent studies in Canada and Hong Kong have demonstrated the estimates of influenza-associated hospitalization derived from Poisson regression models reasonably matched the numbers of patients with laboratory confirmed influenza infections [17], [29]. However, it is extremely difficult to obtain the gold standard data on influenza associated deaths to assess the validity of the statistical models, because patients with influenza infection could have died from secondary bacterial infections and exacerbation of their preexisting conditions [30], [31]. Therefore, their presenting problems may not be directly linked to influenza. Moreover, given the potential lag time between severe complications and primary influenza infection, influenza virus might have become undetectable in these patients in the time of admission. Hence recorded influenza deaths could still seriously underestimate the true numbers of deaths due to influenza even if laboratory tests for influenza are intensively conducted. In this study we performed a simulation study to assess the performance of Poisson regression models. The small biases and RMSE of most estimates may give further evidence to support the validity and reliability of Poisson models.

In this study, we adopted a semi-parametric model with smoothing functions to adjust for potential confounders, whereas most of the other studies just used linear terms of confounding variables in their Poisson models [13], [15]. This semi-parametric model is preferred over the traditional parametric model in that it does not require any pre-determined relationships between the independent and dependent variables and thereby allows us to assess both linear and nonlinear relationships. Although we chose natural spline smoothing functions in this study, there are also other smoothing functions available. However, previous studies have found that having a sufficient number of degrees of freedom is more important than the type of smoothing functions for adequate adjustment of confounding in the semi-parametric model [32]. Therefore, in this simulation study, we mainly focused on the determination of degrees of freedom by an appropriate model selection criterion. Among the four criteria under study, GCV consistently provided the smallest biases and RMSE under the different assumptions of seasonal confounders, particularly when this confounder was assumed to have a high seasonal variation. Our findings were robust to the various assumptions of influenza coefficients in simulation and also to the length of study period. Increase of RMSE was observed when the study period was shorten, which is not surprising as using less data points would increase the variation of estimates.

All the four model selection criteria were developed under the different schemes. PACF measures the autocorrelation of the residuals, whereas both QAIC and QBIC evaluate the relative goodness of fit of a statistical model by quantifying the relative lost of information when a given model is used to describe the reality. Therefore the latter two reflect the tradeoff between accuracy and simplicity, but QAIC penalizes the number of model parameters to a lesser extent than QBIC does. Unlike other criteria, GCV assesses the model validity by cross-validation, i.e. randomly sampling data as training and test datasets to compare the accuracy and variation of prediction. Our findings that GCV outperforms the other criteria in Poisson models are also in line with previous studies on air pollution [25].

We chose the best-fit model based on the adequacy of confounding adjustment in terms of providing reliable estimates for influenza effect. Although many seasonal factors could confound influenza effects on mortality, we only focused on the confounding of long term and seasonal trends of mortality in the present study, as this factor affected our estimates to a greater extent than any other confounders according to our previous experience. In this study the best-fit model was selected by minimizing each of the selection criteria, but the magnitude of their difference was not assessed. Some studies suggested that the models selected under the different criteria might perform equally well if the difference between these criteria were small [33], [34]. However, it is not easy and somewhat arbitrary to define the cutoff points for small difference. Burnham and Anderson (2002) developed a set of cutoff points for AIC to select the models with meaningfully different estimates [33]. Similar thresholds for the BIC value were also introduced by Kass and Raftery [34]. However, so far there are no commonly accepted cutoff points for those selection criteria used in our study. Therefore, we did not take into consideration of the difference magnitude between these values, in order to achieve the simplicity and efficiency in the model selection procedure.

There are several limitations in our study. First we assessed the parameter uncertainty in Poisson regression models based on the data of subtropical city Hong Kong, where influenza seasonality is less clear than that in the temperate regions [16]. Given the well defined winter peaks of influenza in temperate regions, it can be expected that the data from these regions probably required less complex adjustment for seasonal confounders. Nevertheless, the framework developed in our study can still be applied to a wide range of data. Second, we did not separately estimate the effects of different influenza virus subtypes, although previous studies have demonstrated their difference in excess mortality and mutation frequency [35], [36]. Unfortunately, the virus subtype data during the study period are not available to us. Future studies are needed to assess the performance of these model selection criteria in assessing the disease burden associated with each subtype.

By applying Poisson regression models to an empirical dataset of influenza hospitalization, we demonstrate that our findings can be generalized to other health outcomes. The best-fit models were validated by comparing the estimates of age-specific excess hospitalization rates with the observed rates in a pediatric cohort undergoing intensive laboratory tests for influenza infections. Consistent with the findings of our mortality simulated study, GCV criterion outperformed QAIC, QBIC and PACF with smaller biases and RMSE. Given the enormous cost in money and manpower by such a prospective cohort study, statistical modeling is relatively easier to conduct and able to provide reliable estimates for influenza associated disease burden.

In conclusion, our results suggested that the GCV criteria should be recommended for selection of the best-fit model in the future disease burden studies using Poisson models. Standardization of this modeling procedure shall increase the reliability of estimates and facilitate the comparison across countries or regions.

## Supporting Information

Figure S1 **Sensitivity analysis by influenza coefficient.** Bias and RMSE of influenza coefficient estimates from the models selected by different criteria, (A, B) when the simulation coefficient for influenza was fixed to 0.1 and (C, D) when the simulation coefficient for influenza fixed to 0.5. Abbreviations: QAIC, quasi-Akaike information criterion; QBIC, quasi-Bayesian information criterion; PACF, partial autocorrelation function; GCV, generalized cross validation; RMSE, root-mean-square error
(TIF)

Figure S2 **Sensitivity analysis by study period.** Bias and RMSE of influenza coefficient estimates from the models selected by different criteria, (A, B) during the study period of 2006 to 2008, and (C, D) the study period of 2003 to 2008. Abbreviations: QAIC, quasi-Akaike information criterion; QBIC, quasi-Bayesian information criterion; PACF, partial autocorrelation function; GCV, generalized cross validation; RMSE, root-mean-square error;
(TIF)

Figure S3 **Sensitivity analysis by lag effect.** Bias and RMSE of influenza coefficient estimates from the models selected by different criteria, for (A, B) the lag effect of 1 week, (C, D) the lag effect of 2 weeks and (E,F) the lag effect of 3 weeks. Abbreviations: QAIC, quasi-Akaike information criterion; QBIC, quasi-Bayesian information criterion; PACF, partial autocorrelation function; GCV, generalized cross validation; RMSE, root-mean-square error;
(TIF)

Figure S4 **Weekly numbers of observed and fitted hospitalization by age group.** The fitted hospitalization data were derived from the best-fit models selected by the generalized cross validation (GCV) criterion.
(TIF)
