# Supplementary figures and images for: Model Selection in Time Series Studies of Influenza-Associated Mortality
Source: PLoS One. 2012 Jun 20;7(6):e39423. doi: 10.1371/journal.pone.0039423 (PMC3380027; doi:10.1371/journal.pone.0039423)

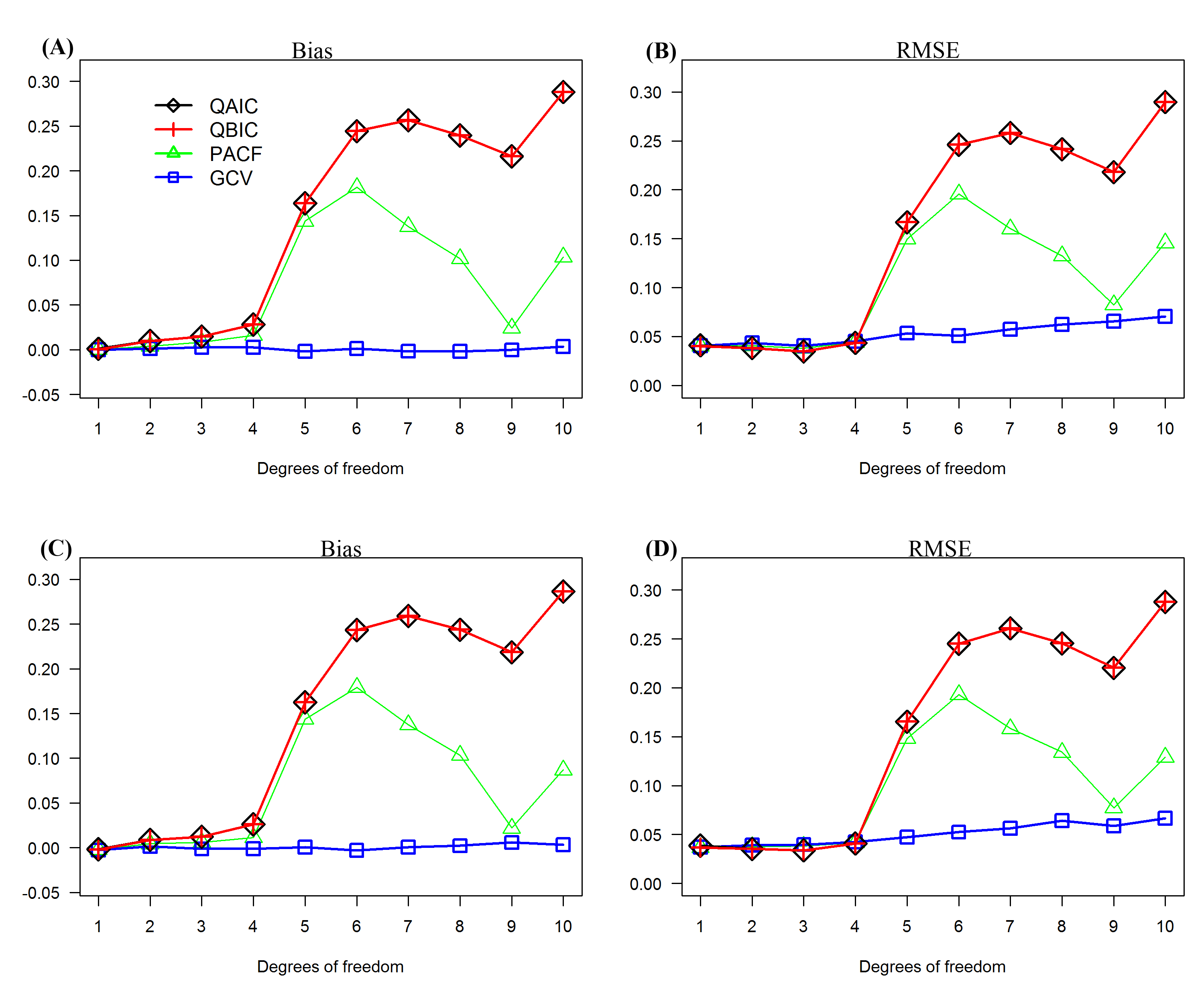

Supplement: Figure S1 — Sensitivity analysis by influenza coefficient. Bias and RMSE of influenza coefficient estimates from the models selected by different criteria, (A, B) when the simulation coefficient for influenza was fixed to 0.1 and (C, D) when the simulation coefficient for influenza fixed to 0.5. Abbreviations: QAIC, quasi-Akaike information criterion; QBIC, quasi-Bayesian information criterion; PACF, partial autocorrelation function; GCV, generalized cross validation; RMSE, root-mean-square error (TIF) [file pone.0039423.s001.tif]

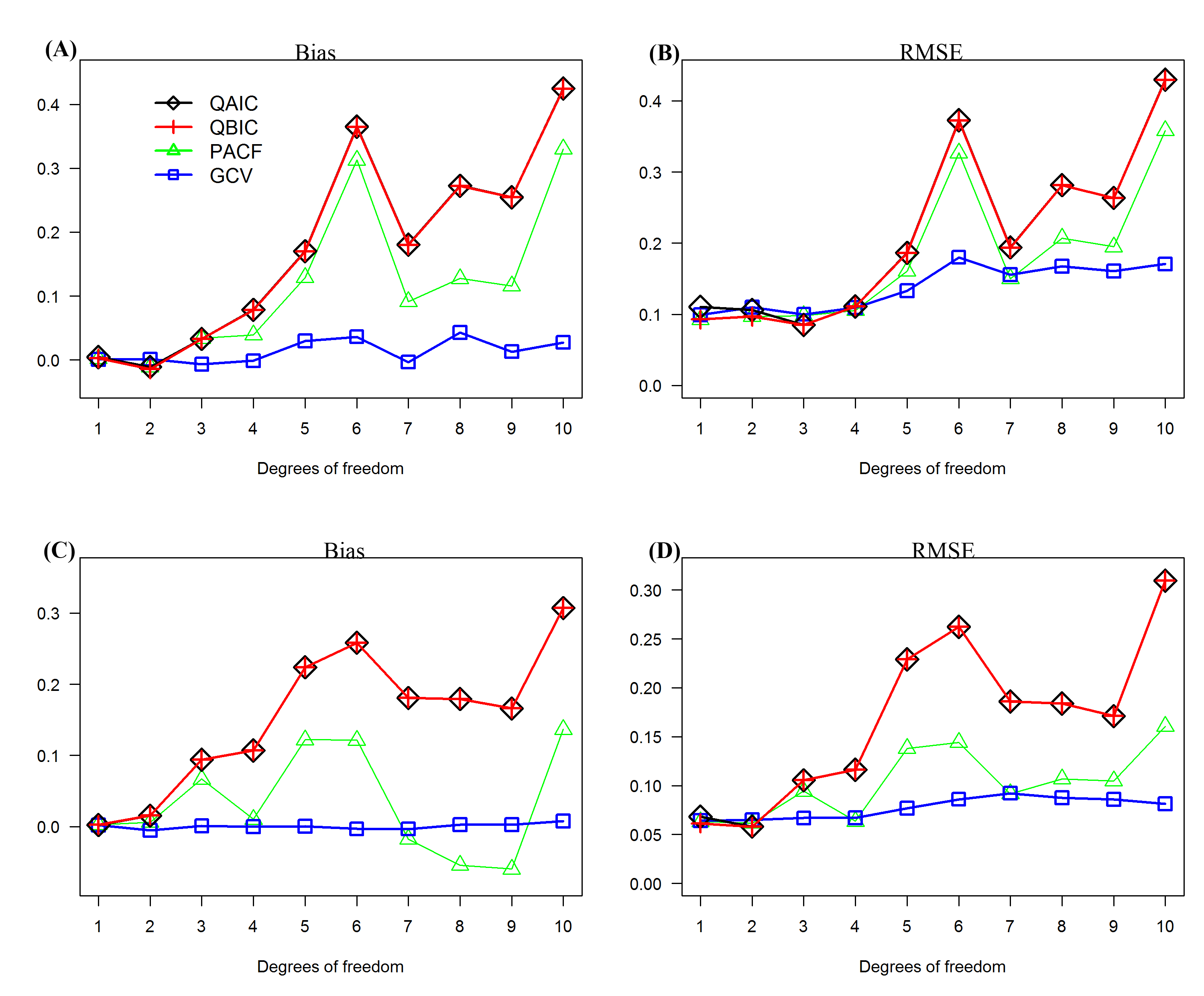

Supplement: Figure S2 — Sensitivity analysis by study period. Bias and RMSE of influenza coefficient estimates from the models selected by different criteria, (A, B) during the study period of 2006 to 2008, and (C, D) the study period of 2003 to 2008. Abbreviations: QAIC, quasi-Akaike information criterion; QBIC, quasi-Bayesian information criterion; PACF, partial autocorrelation function; GCV, generalized cross validation; RMSE, root-mean-square error; (TIF) [file pone.0039423.s002.tif]

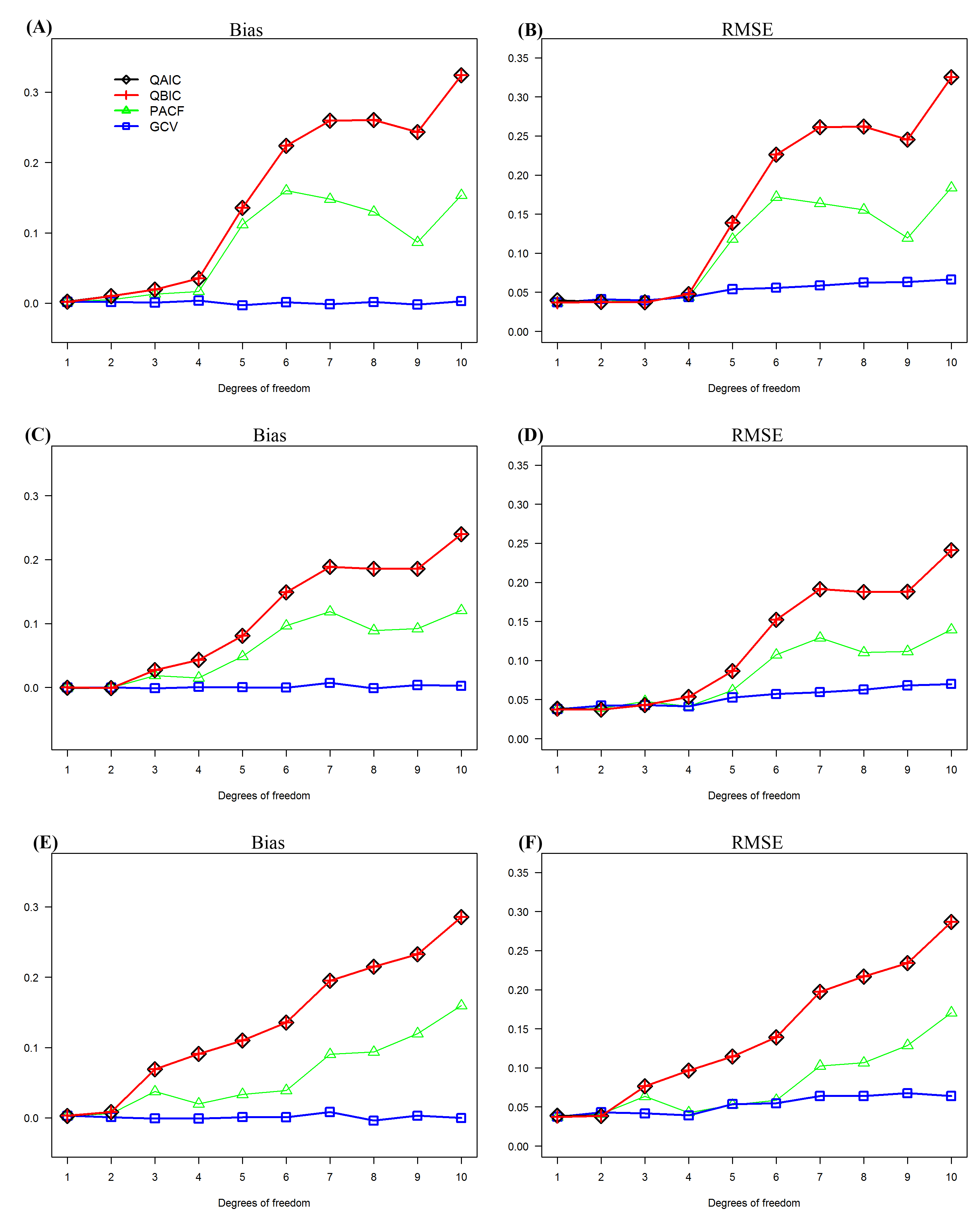

Supplement: Figure S3 — Sensitivity analysis by lag effect. Bias and RMSE of influenza coefficient estimates from the models selected by different criteria, for (A, B) the lag effect of 1 week, (C, D) the lag effect of 2 weeks and (E,F) the lag effect of 3 weeks. Abbreviations: QAIC, quasi-Akaike information criterion; QBIC, quasi-Bayesian information criterion; PACF, partial autocorrelation function; GCV, generalized cross validation; RMSE, root-mean-square error; (TIF) [file pone.0039423.s003.tif]

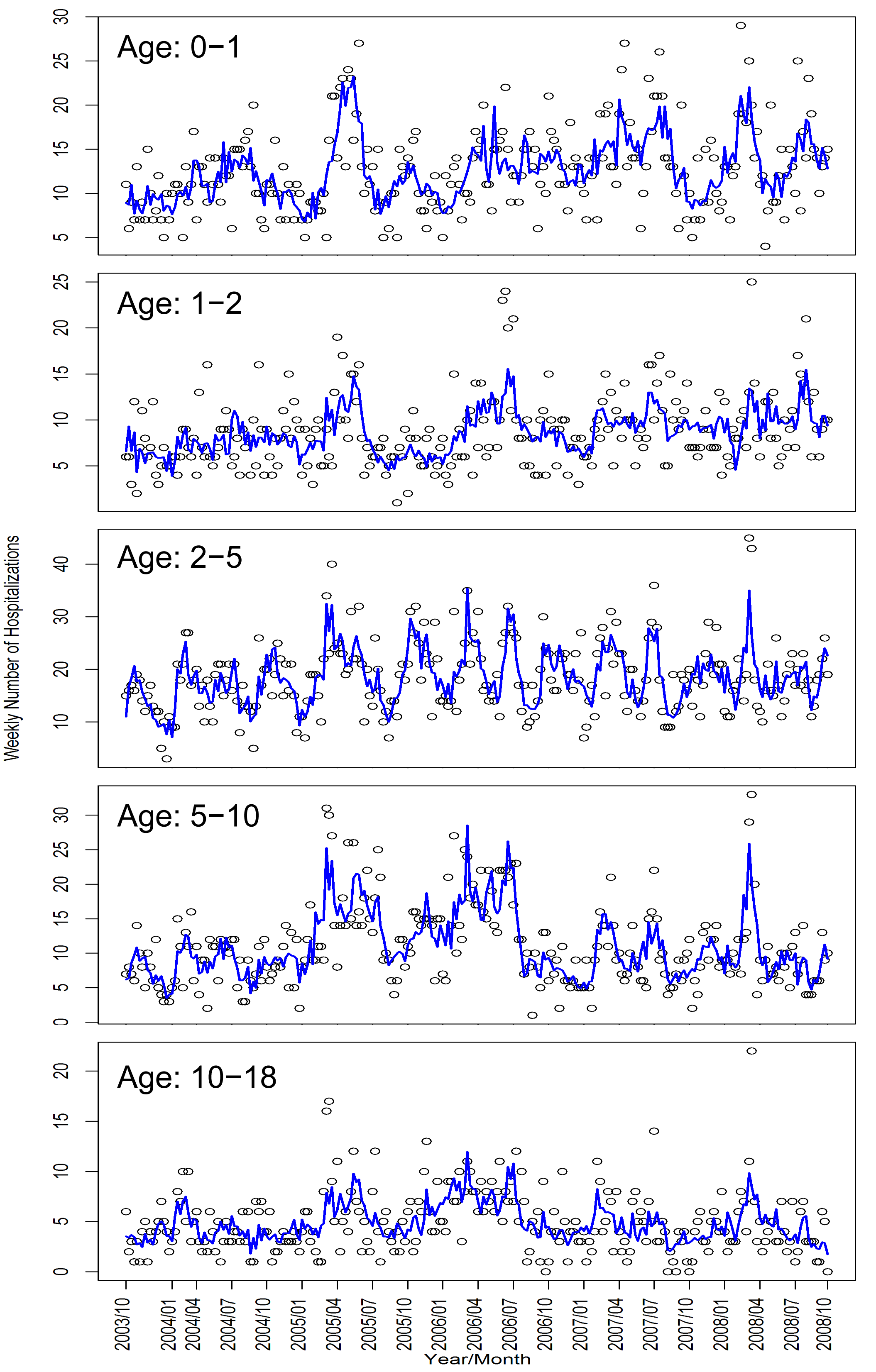

Supplement: Figure S4 — Weekly numbers of observed and fitted hospitalization by age group. The fitted hospitalization data were derived from the best-fit models selected by the generalized cross validation (GCV) criterion. (TIF) [file pone.0039423.s004.tif]
